# Wild Mice As Bountiful Resources of Novel Genetic Variants for Quantitative Traits

**DOI:** 10.2174/1389202911314040001

**Published:** 2013-06

**Authors:** Akira Ishikawa

**Affiliations:** Laboratory of Animal Genetics, Division of Applied Genetics and Physiology, Graduate School of Bioagricultural Sciences, Nagoya University, Nagoya, Aichi 464-8601, Japan

**Keywords:** Classical inbred strain, Genetic resources, Natural variation, Quantitative trait, QTL, Wild mice.

## Abstract

Most traits of biological importance, including traits for human complex diseases (e.g., obesity and diabetes), are continuously distributed. These complex or quantitative traits are controlled by multiple genetic loci called QTLs (quantitative trait loci), environments and their interactions. The laboratory mouse has long been used as a pilot animal model for understanding the genetic architecture of quantitative traits. Next-generation sequencing analyses and genome-wide SNP (single nucleotide polymorphism) analyses of mouse genomes have revealed that classical inbred strains commonly used throughout the world are derived from a few fancy mice with limited and non-randomly distributed genetic diversity that occurs in nature and also indicated that their genomes are predominantly *Mus musculus domesticus* in origin. Many QTLs for a huge variety of traits have so far been discovered from a very limited gene pool of classical inbred strains. However, wild *M. musculus* mice consisting of five subspecies widely inhabit areas all over the world, and hence a number of novel QTLs may still lie undiscovered in gene pools of the wild mice. Some of the QTLs are expected to improve our understanding of human complex diseases. Using wild *M. musculus* subspecies in Asia as examples, this review illustrates that wild mice are untapped natural resources for valuable QTL discovery.

## INTRODUCTION

Most traits of biological importance, including traits for human complex diseases (e.g., obesity and diabetes) and agricultural and livestock products (e.g., crop yield and meat quality), are continuously distributed. These traits are called complex or quantitative traits and are controlled by multiple genetic loci referred to as QTLs (quantitative trait loci), environments and their interactions. The laboratory mouse has been long and widely used as a pilot model organism for understanding the genetic architecture of quantitative traits because of its small body size, short gestation period (approximately three weeks), cost-effective rearing and extensive genome information that is freely available [1, 2]. Several thousand QTLs for a huge variety of quantitative traits have been mapped to chromosomal regions using inbred mouse strains [3]. In 2002, the whole-genome sequence data of C57BL/6J, a classical laboratory inbred strain, were released and it was revealed that ~99% of mouse genes have human homologues [4]. This finding further reinforces the role of the mouse as the premier model for elucidating the genetic and molecular basis of human complex diseases and other quantitative traits.

Next-generation sequencing of mouse genomes of 13 classical inbred strains and four wild-derived inbred strains originating directly in wild-caught mice, belonging to three *Mus musculus* subspecies (*M*.* m*. *castaneus*, *M*.* m*. *domesticus* and* M*.* m*. *musculus*) and *Mus spretus* taxa, revealed 129,260,574 SNPs (single nucleotide polymorphisms), 711,920 structural variants (insertions, deletions, copy number gains and others) and candidate variants at 718 QTLs for diseases and physiological traits [5]. On the other hand, genome-wide SNP and VINO (variable intensity oligonucleotide) analyses of 100 classical inbred mouse strains, 62 wild-derived inbred strains originating in *M*.* m*. *castaneus*, *M*.* m*. *domesticus*, *M*.* m*. *molossinus* and* M*.* m*. *musculus* subspecies and 36 wild-caught *M*.* m*. *castaneus*, *M*.* m*. *domesticus* and* M*.* m*. *musculus* mice revealed that (1) the classical inbred strains are derived from a few fancy mice with limited haplotype diversity, (2) most of their genome is derived from *M. m. domesticus* (94.3%), *M. m. musculus* (5.4%) and *M. m. castaneus *(0.3%), and (3) approximately 35% of the genome of the classical inbred strains is IBD (identical by descent) [6]. These findings suggest that when a QTL mapping experiment is performed in a backcross or F2 intercross population between two classical inbred strains, no QTL can be identified in the IBD regions and that even if QTLs can be mapped, they will be loci discovered from a very limited gene pool of a few fancy mice. In other words, the QTLs mapped so far using classical inbred strains must be just the tip of the iceberg. Hence, a number of novel QTLs, some of which are obviously expected to improve our understanding of the genetic basis of complex diseases and other quantitative traits, may be buried in an untapped natural resource of wild mice inhabiting the earth. In this review, I illustrate the importance of wild mice as a natural resource for valuable QTL discovery with focus on two Asian mouse subspecies, *M. m. castaneus* and *M. m. molossinus*, because (1) the genome contribution of *M. m. castaneus* to classical inbred mice is the smallest among the four subspecies as described above, (2) *M. m. molossinus* has a unique historical origin as I will describe below, and (3) much information on two European subspecies, *M. m. domesticus* and* M. m. musculus*, and on *M. spretus*, which has been long and widely used as a non-*M*. *musculus *mouse model, has been made available, for example, in the Mouse Genome Database (MGD) [3].

## GEOGRAPHICAL ORIGIN OF WILD MICE

Five subspecies of *Mus musculus*, i.e., *M. m. domesticus*, *M. m. musculus*, *M. m. castaneus*, *M. m. molossinus* and* M. m. bactrianus*, have evolved and radiated outward within the past million years from a common ancestor inhabiting the northern area of the Indian subcontinent, an area believed to be part of the ancestral range of the *Mus musculus* subspecies complex [7, 8]. Nowadays, they inhabit areas all over the world.

*M. m. domesticus* is distributed in Western Europe, Africa and the Near East, and it has been transported by humans to America and Australia. *M. m. musculus* inhabits areas from Eastern Europe to northern Asia. Compared to these two European subspecies, the remaining three subspecies are distributed in relatively restricted areas in Asia. *M. m. castaneus* is mainly distributed in Southeast Asia. *M. m. molossinus* is found only in Japan and this subspecies has uniquely arisen by natural hybridization of *M. m. musculus* in China and *M. m. castaneus* in Southeast Asia [7, 8]. Interestingly, the *M. m. musculus* genome regions found in the classical inbred strains have been reported to be mostly derived from *M. m. molossinus* [6]. *M. m. bactrianus* inhabits areas around India [8], but it is not known whether this species has contributed to the genome of classical strains.

Although *M. m. domesticus* and *M. m. musculus* subspecies do not live in sympatry as described above, they can be easily crossed with each other in laboratories. The hybrid males obtained often become sterile [9]. In contrast, a high proportion of gene exchanges has recently been found between natural populations of these two subspecies [10]. Similarly, hybrid males between the other subspecies are likely to have developed some kinds of reproductive isolation mechanisms. Presenting examples of them is out of the scope of this review.

## EXAMPLE OF *M*. *m. castaneus*

CAST/EiJ is an inbred strain derived from wild *M. m. castaneus* mice in Thailand. This strain has been frequently used all over the world. Unfortunately, it has been revealed that approximately 12% of its genome is contaminated by other subspecies or classical inbred strains [6]. Hence, I omitted mentioning CAST/EiJ in this review.

One of my colleagues captured live a pair of adult wild *M. m. castaneus* mice in Los Baños, Luzon Island, the Philippines in June 1994. The mice captured were introduced into my laboratory and immediately mated with each other. Their descendants were mated with the C57BL/6J inbred strain to develop a QTL mapping population of 387 backcross mice. The wild mice have only 60% of the body weight of C57BL/6J. Using the backcross population, I performed genome-wide QTL analysis and found 24 QTLs for body weight at 3-10 weeks of age and for body weight gains at 3-6 weeks and 6-10 weeks of age on 13 mouse chromosomes [11-13]. The 24 QTLs identified have main effects and/or epistatic interaction effects on the traits, and several loci also have sex-specific effects. Among the 24 QTLs, the most potent QTL (named *Pbwg1*) on chromosome 2 increases its effect linearly with increasing age and accounts for 3.7-12.1% of the total phenotypic variance depending on the age examined. As expected, the *Pbwg1 *allele derived from the wild *M. m. castaneus* mouse retards growth [13]. To confirm the presence of *Pbwg1* and to narrow down its chromosomal location, I developed a congenic strain, named B6.Cg-*Pbwg1*, with an *M. m. castaneus* genome region of approximately 44 Mb on the C57BL/6J genetic background [14]. I also developed more than 20 subcongenic strains with overlapping and non-overlapping genome regions from an F2 intercross of the B6.Cg-*Pbwg1* congenic strain and the C57BL/6J strain (all congenic and subcongenic strains deposited in RIKEN BioResource Center, Japan (http:// www.brc.riken.jp/lab/animal/)) [14, 15]. 

Obesity is characterized by excessive fat accumulation in adipose tissue and other organs. Human obesity is now a major health concern worldwide because it is an important predisposing factor for chronic diseases such as metabolic syndrome, cardiovascular disease and cancer [16]. In humans, body mass index (weight in kg/height in meters squared) is used as a measurement of body fat in clinical and epidemiologic studies. In mice, the weight of white fat depots such as gonadal fat pads has been long and widely used as an indicator because fat depots are relatively easy to dissect out and are highly correlated to total body fat [1]. Genetic analyses using the B6.Cg-*Pbwg1 *congenic strain and subcongenic strains derived from B6.Cg-*Pbwg1* revealed an obesity QTL within an approximately 8.8-Mb region between two microsatellite markers, *D2Mit270* and *D2Mit472*, on chromosome 2. The wild-derived QTL allele prevented obesity in mice fed both standard (5.1% crude fat and 3.45 Kcal/g energy) and high-fat (24% and 4.73 Kcal/g) diets. For 13-week-old male mice fed a standard diet, the total weights of inguinal, gonadal and retroperitoneal white fat pads were 0.790 ± 0.004 (least-squared mean ± standard error (SE)) g in the B6.Cg-*Pbwg1* congenic strain and 0.641 ± 0.007 g in the B6.Cg-*Pbwg1*/SR8 subcongenic strain. Both means were significantly lower than that of C57BL/6J males (1.043 ± 0.009 g). Similarly, when mice were fed a high-fat diet for 7 weeks from 6 to 13 weeks of age, total fat weights were 1.745 ± 0.081 g in B6.Cg-*Pbwg1* and 1.221 ± 0.098 g in B6.Cg-*Pbwg1*/SR8, and both were significantly lower than that of C57BL/6J (3.100 ± 0.083 g) [15]. In addition, the wild-derived QTL allele decreased body weight and serum levels of glucose and triglyceride in mice fed a standard diet. Identification of a causative gene for the obesity-resistant QTL discovered from the wild *M. m. castaneus* mouse may play an important role in elucidation of the molecular mechanisms involved in adipogenesis and obesogenesis.

Furthermore, QTL analysis in a population of 269 F2 intercross mice between B6.Cg-*Pbwg1 *and C57BL/6J strains revealed several closely-linked QTLs affecting body weight gain and body composition traits [14, 17]. Among the linked QTLs, I uniquely localized an overdominant QTL (named *Pbwg1.10*) causing heterosis for body weight at 6 weeks of age, within an approximately 21-Mb confidence interval. The 6-week body weight of mice heterozygous for the *Cas* allele derived from wild *M. m. castaneus* mice and the *B6* allele derived from C57BL/6J at a microsatellite marker nearest *Pbwg1.10 *was 20.1 ± 0.1 (least-squared mean ±SE) g. This value was significantly higher than those of two types of homozygotes, *Cas*/*Cas* (19.5 ± 0.2 g) and *B6*/*B6 *(19.3 ± 0.2 g). The degree of dominance was 6.6 [17]. Heterosis is a genetic phenomenon necessary for animal breeding as well as plant breeding. Two prominent hypotheses explaining heterosis have been advocated so far. One is a dominance hypothesis and the other is an overdominance hypothesis. However, it is not known whether specific loci exhibit overdominance effects or whether heterozygosity itself confers heterosis in a genome-wide manner [18]. Recently, the *SFT* (single flower truss) gene has been identified as the first overdominant gene responsible for heterosis of yield in tomato [19]. In contrast, no such gene has been cloned in animals. Hence, the molecular mechanisms of heterosis are not well understood. Since the overdominant QTL discovered from the wild *M. m. castaneus* mouse has undergone natural selection, it will be interesting to see how the observed heterotic phenotype contributes to biological evolution because body weight is one of the fitness traits.

## EXAMPLE OF *M. m. molossinus*

“Chingen-sodategusa” is the oldest Japanese guidebook for fancy mice published in 1787. Various kinds of visible mouse mutants, such as coat color and behavior, their modes of inheritance and their breeding methods are described in this book [20]. The fancy mice often became subjects of Japanese fine arts such as porcelain bowls and “ukiyoe”, Japanese Edo woodblock prints, in the Edo period (1603-1867). The late Kyoji Kondo, a professor emeritus at my laboratory, Nagoya University, developed unique inbred strains from native Japanese fancy mice for the first time in Japan. For example, in 1944, he developed the KK (Kasukabe-K) strain from a Japanese dealer stock in Kusakabe, Saitama Prefecture, a rural area believed to be the region where farmers had bred and sold fancy mice since the Edo period [21]. KK is now one of world-famous mouse models for type 2 diabetes [22]. In 1955, he established the NC strain from a cinnamon coat color colony of Japanese fancy mice called Nishiki-Nezumi. The NC mouse is internationally used as a model of atopic dermatitis [23]. Since the 1960s, Professor Kondo and his students have started to breed Japanese wild *M. m. molossinus* mice captured live in the suburbs of Nagoya University, Aichi Prefecture and established some inbred strains such as MOA and MOM [21]. Crosses between females of the DDK strain and males of classical inbred strains incur early embryonic lethality known as the DDK syndrome, owing to incompatibility between a maternal DDK factor and a paternal gene, both of which map to the *Om* (ovum mutant) locus on chromosome 11 [24]. Interestingly, when DDK females are mated with males of the MOM strain as well as the CASP strain derived from *M. m. castaneus*, the paternal MOM and CASP genes are fully compatible with the maternal DDK factor [25].

Inspired by Kondo’s pioneering works described above, some Japanese geneticists have developed wild-derived inbred strains of *M. m. molossinus*. For example, five inbred strains (KOR1/Stm, KOR5/Stm, KOR7/Stm, AIZ/Stm and MAE/Stm) have been established from pairs of Japanese wild mice trapped in the Tohoku area [21]. During their breeding, several mutations were spontaneously discovered, including mutations responsible for hyperlipidemia and arteriosclerosis [26], atopic dermatitis [27], microphthalmia, dominant white spots, sebaceous gland abnormalities and audible song-like vocalization [21]. In addition, MSM/Ms has been established from wild mice captured in Mishima, Shizuoka Prefecture [28] and it displays large phenotypic differences in many quantitative traits, such as traits for growth, energy metabolism and behavior, compared with C57BL/6J (NIG Mouse Phenotype Database, http://molossinus.lab.nig.ac.jp/phenotype/index.html). Therefore, many novel QTLs that are not present in the gene pool of classical inbred strains may be discovered from the wild-derived strains established.

## ADVANTAGES AND DISADVANTAGES OF USING WILD MICE

There are some advantages of using wild mice for mapping QTLs. One advantage is that many polymorphic microsatellite markers can be easily selected. I was able to select a total of 88 microsatellite markers distributed over all autosomes and X chromosome from approximately 200 microsatellite markers listed in MGD [11]. All alleles of these markers were able to be completely distinguished between Philippine wild *M. m. castaneus* mice and C57BL/6J mice because the genome of C57BL/6J is mostly derived from *M*.* m*. *domesticus*. In addition, I did not require any SNP markers for fine mapping QTLs [14, 15]. Since it is easier and cheaper to genotype microsatellite markers than to genotype SNP markers, there is still merit in using microsatellite markers for QTL mapping. However, there will be a case that two or more marker alleles are segregating within the wild mice. Nevertheless, QTL analysis can be performed, as I have carried out, with the assumption of a di-allelic system, i.e., the segregating markers were treated as fixed markers [11].

Another advantage is that genome-wide association studies (GWAS) using wild mice may provide a high mapping resolution of a QTL like human GWAS because wild mice have undergone a great number of recombination events [29]. Mapping resolution affects the number of genes that need be tested as positional candidate genes for a QTL. The low mapping resolution obtained by a conventional QTL analysis in a backcross or F2 intercross between two inbred mouse strains provides a large confidence interval of the QTL, often tens of megabases, that contains hundreds of genes. Therefore, it will be too time-consuming and costly to identify candidates from so many genes. The high mapping resolution obtained by GWAS would need fewer genes to be tested. However, in practice, it would be difficult to perform GWAS in wild mice for the reasons described in [29]. Briefly, thousands of unrelated wild mice are required and whole-genome sequencing analysis is needed to obtain genetic variation within the wild mice. To overcome these problems, as was done in the studies reviewed here, it would be better for QTL mapping to use wild mice carrying only a single haplotype or a few haplotypes that have been obtained from a wild population for mating them with mice of an inbred strain to develop a QTL mapping population, but the mapping resolution obtained would be low.

A major disadvantage of using wild mice for mapping QTLs is that a large number of mutations such as SNPs, insertions and deletions are included in the wild mouse genome. For example, I have very recently sequenced all exons of genes on the wild-derived genome region of the B6.Cg-*Pbwg1 *congenic strain. A large number of synonymous SNPs and non-synonymous SNPs were found (unpublished data).

The road from a QTL to a candidate gene is generally considered to be a long one, as reviewed in [30]. However, when I focus on a QTL, the causative gene for which affects the amount of gene expression, I consider the road to be time-consuming but not a hard one when modern-day technologies such as next-generation sequencing analysis and computational tools are used. Briefly, my ongoing process to identify a candidate gene for the obesity-resistant QTL is shown as an example. Congenic and subcongenic analyses are performed to physically define the genome region of the QTL as small as possible. Next, RNA-sequencing analysis is carried out in a subcongenic strain with the smallest QTL region and its background strain. The genes differentially expressed in the two strains are considered to be candidate genes, and the number of the genes will therefore not be small. In addition, genes on the subcongenic region having non-synonymous SNPs on the exons can become candidate genes, the number of which will not be small as in my case. These candidate genes can be easily prioritized by use of computational tools such as Endeavour [31] and SIFT [32]. Endeavour prioritizes candidate genes that are differentially expressed by using seed genes associated with human diseases as prioritization criteria [31]. SIFT predicts tolerated and deleterious substitutions for non-synonymous SNPs based on the evolutionary conservation of the amino acids within protein families [32]. Furthermore, the number of candidate genes can be reduced by testing associations between gene expression levels and trait values in an F2 segregating population between the subcongenic and background strains. In fact, I was able to identify a few candidate genes for the obesity-resistant QTL (unpublished data). This number of candidate genes must be a realistic number for carrying out validation experiments of the candidate genes using knockout mice.

## CONCLUSION

Wild mice of *M. m. castaneus* and *M. m. molossinus* subspecies displayed unique phenotypes of many quantitative traits compared to those of classical inbred strains. In fact, unique QTLs affecting obesity resistance and heterosis for body weight have been discovered from the wild *M. m. castaneus *mice. These QTLs are probably novel because no loci associated with these and related traits have been reported on an approximately 25-Mb genome region on chromosome 2 harboring the QTLs (QTL Viewer, http://www.genomics.liv.ac.uk/tryps/QTLDatabase/). In addition to these wild mice, many other colonies or inbred strains derived from different populations of these subspecies and other *M*.* musculus* subspecies have been established and are listed in databases such as RIKEN BioResource Center (http://www.brc.riken.jp/lab/animal/) and the Jackson Laboratory (http://research.jax.org/grs/type/wild/index.html). It is expected that a number of novel QTLs still lie undiscovered in the gene pools of these wild mice.

Importantly, genes at the QTLs discovered from the wild mice contain the underlying causal variants of quantitative traits that have undergone natural selection during the evolution of this species. Such natural variant genes must be obviously different in nature from genetically engineered and artificially induced genes that do not have evolutionary histories at all. Hence, natural variant genes uncovered from wild mice are most certainly model genes of human complex diseases because human disease genes are naturally occurring genes. Information obtained from exploring the natural variant genes of wild mice will not only help us to understand the genetic and molecular architecture of human complex diseases but will also lead to efficient prediction and prevention of the diseases. Wild mice are thus bountiful untapped natural resources for valuable QTL discovery.
